# Fifteen-Year Follow-Up of Nanos Neck-Preserving Hip Arthroplasty: An Observational Retrospective Study

**DOI:** 10.3390/jfmk10040389

**Published:** 2025-10-05

**Authors:** Giuseppe Rovere, Davide Luziatelli, Sandro Luziatelli, Gianluca Polce, Pierfrancesco Pirri, Vincenzo De Luna, Francesco Liuzza, Pasquale Farsetti, Fernando De Maio

**Affiliations:** 1 Section of Orthopaedics and Traumatology, Department of Clinical Science and Translational Medicine, University of Rome “Tor Vergata”, 00133 Rome, Italypierfrancesco.pirri@gmail.com (P.P.); videluna@hotmail.com (V.D.L.); liuzzafrancesco@gmail.com (F.L.); farsetti@uniroma2.it (P.F.);; 2 Villa Letizia, Strada Statale 80, 25/b, 67100 L’Aquila, Italy; 3 Orthopaedics and Traumatology Unit, Celio Military Hospital, 00100 Rome, Italy; polce.gianluca@icloud.com

**Keywords:** total hip arthroplasty (THA), neck-preserving hip arthroplasty, Nanos prosthesis, femoral neck preservation, bone stock conservation

## Abstract

**Introduction:** Neck-preserving total hip arthroplasty (THA) has gained interest for conserving bone stock, restoring biomechanics, and facilitating revision surgery. The Nanos^®^ femoral stem, designed for metaphyseal fixation while preserving the femoral neck, represents a reliable alternative to conventional THA. This study reports 15-year clinical and radiographic outcomes of the Nanos implant. **Materials and Methods:** We retrospectively reviewed 53 patients (35 males, 18 females) who underwent THA with the Nanos stem between 2008 and 2010. Patients were stratified into two groups according to age: <50 years (*n* = 24) and ≥50 years (*n* = 29). The primary diagnosis was osteoarthritis (95%), with a few cases of avascular necrosis or dysplasia. Clinical evaluation included the Harris Hip Score (HHS) and the Western Ontario and McMaster Universities Arthritis Index (WOMAC). Radiographic assessment focused on implant stability, osteolysis, and heterotopic ossifications. Kaplan–Meier survival analysis was performed with revision for any reason as the endpoint. **Results:** At 15 years of follow-up, both groups showed significant improvement (*p* < 0.001). In patients <50 years, HHS increased from 53.3 to 94.8 and WOMAC decreased from 79.9 to 3.5. In patients ≥50 years, HHS improved from 47.5 to 95.2 and WOMAC from 81.5 to 3.2. Radiographs confirmed stable fixation without osteolysis. Complications included two dislocations and one cortical perforation requiring revision. Kaplan–Meier survivorship at 15 years was 100% (<50) and 96.6% (≥50). **Conclusions:** The Nanos stem provided excellent long-term outcomes with low complication and revision rates. It should be considered one of several reliable short-stem options for younger, active patients, offering durable function while preserving bone stock.

## 1. Introduction

Total hip arthroplasty (THA) has undergone significant evolution in recent decades, driven by continuous advancements in surgical techniques, implant design, and rising expectations from both patients and surgeons [1,2,3]. Among the primary goals of modern THA is the preservation of bone stock, which has become a central principle, particularly in younger and more active patients.

Conventional femoral stems typically require substantial resection of the femoral neck to achieve stable fixation. However, this approach compromises native bone stock, posing challenges for potential future revision procedures [4,5,6]. In response, bone-conserving implants have been developed to minimize bone loss during initial surgery. Among these, femoral neck-preserving prostheses such as the Nanos^®^ stem (Smith & Nephew, Tuttlingen, Germania) represent a paradigm shift in hip arthroplasty [7,8].

By preserving the femoral neck, these implants maintain key anatomical and biomechanical structures, facilitating restoration of native hip biomechanics, including femoral offset, limb length, and anteversion. This contributes to optimal muscle tension, joint stability, and more physiological load distribution along the proximal femur [9,10,11,12,13,14]. Additionally, the preserved cortical and cancellous bone of the femoral neck provides reliable metaphyseal support, enhancing implant stability [15,16].

Biomechanically, femoral neck preservation promotes a more natural transfer of forces across the joint, mitigating the risk of stress shielding and proximal bone resorption, which are common with traditional long stems [17,18,19,20,21]. Modern designs, such as the trapezoidal-conical geometry of the Nanos stem, further optimize load transfer and initial stability [22,23].

Moreover, neck-preserving THA aligns with the principles of minimally invasive surgery (MIS), reducing surgical trauma, preserving soft tissue integrity, and facilitating quicker recovery. This approach fits seamlessly with current trends toward patient-centered, tissue-sparing techniques [24,25,26,27].

While short-term outcomes of neck-preserving stems have shown promising results in terms of pain relief, function, and patient satisfaction [28,29,30], long-term evidence is still limited. Given the increasing use of these implants, especially in younger populations, evaluating their durability and long-term performance is essential [31,32,33,34,35,36,37,38,39,40].

Given the lack of a conventional THA control group in our cohort, results were interpreted in the context of previously published comparative studies and registry data. This approach allows benchmarking of the Nanos prosthesis against established conventional stems, thereby strengthening the external validity of our findings.

This study aims to assess the long-term clinical and radiographic outcomes of the Nanos femoral neck-preserving prosthesis over a 15-year follow-up period. The objective is to provide robust data on implant survivorship, functional recovery, and complication rates, offering valuable insight into its viability for younger, active patients who may require future revision surgery.

## 2. Materials and Methods

### 2.1. Ethical Aspects

This study was conducted in accordance with the principles of the Declaration of Helsinki. As a retrospective observational study, formal ethical approval was not required. All patients provided written informed consent for the use of their clinical and radiographic data for research purposes. Data anonymity and confidentiality were ensured throughout the study.

### 2.2. Eligibility Criteria

Patients were included if they underwent total hip arthroplasty using the Nanos neck-preserving femoral stem between January 2008 and December 2010 and had a minimum follow-up of 15 years. Inclusion criteria were diagnosis of primary or secondary osteoarthritis, age between 35 and 65 years at the time of surgery, and the availability of complete clinical and radiographic records. Patients with previous hip surgeries, significant deformities requiring custom implants, active infection, or systemic conditions contraindicating THA were excluded. All patients provided informed consent for data use in research.

### 2.3. Description of the Experimental Stage

This retrospective study included 53 patients who underwent total hip arthroplasty with the Nanos neck-preserving femoral stem (Smith & Nephew, Tuttlingen, Germania) between 2008 and 2010. All procedures were performed by the same senior orthopedic surgeon using a minimally invasive posterior approach (Gibson-Moore technique), with the patient positioned in lateral decubitus. The study population consisted of 35 males and 18 females, with a mean age of 50.7 years at the time of surgery. The primary indication was osteoarthritis (95%), with the remaining cases including avascular necrosis, developmental dysplasia of the hip, and sequelae of acetabular fractures.

The Nanos^®^ femoral component is a short, neck-preserving stem with trapezoidal-conical geometry, designed for primary metaphyseal fixation while maintaining the femoral neck. It is manufactured from titanium alloy (Ti6Al4V) with a proximal porous titanium and hydroxyapatite coating to promote osseointegration, while the distal portion is polished to minimize stress shielding. This design facilitates restoration of femoral offset and anteversion, enabling a more physiological load transfer and reducing proximal bone resorption. The acetabular component was the R3™ porous coated hemispherical cup (Smith & Nephew, Tuttlingen, Germany). The shell is made of Ti6Al4V alloy with a STIKTITE™ porous coating to enhance initial fixation and promote osseointegration. Sizes range from 48 to 68 mm in 2 mm increments, and the shell can be configured with 0 or 3 screw holes to allow adjunctive screw fixation when necessary. The system supports multiple liner options, including highly cross-linked polyethylene (XLPE) and ceramic liners, with a locking mechanism designed to ensure both axial and rotational stability.

In our series, the chosen bearing surface was ceramic-on-polyethylene (CoP), using highly cross-linked polyethylene liners.

Femoral heads ranged from 28 to 46 mm in diameter, with ceramic 36 mm heads being the most frequently used, in order to optimize joint stability, range of motion, and wear resistance.

Postoperative follow-up was scheduled at 3, 6, and 12 months, and annually thereafter. Each follow-up included clinical examination, functional assessment, and radiographic analysis. Range of motion (ROM) was evaluated in terms of flexion, extension, abduction, and rotation. Leg length discrepancy (LLD) was measured, and patients were queried regarding audible joint sounds (e.g., squeaking).

Clinical outcomes were assessed using the Harris Hip Score (HHS) and the Western Ontario and McMaster Universities Arthritis Index (WOMAC). Radiographic assessments included standard anteroposterior (AP) views of the pelvis and affected hip. Key parameters analyzed were acetabular cup inclination, implant position and stability, presence of osteolysis (based on Gruen zones), and the extent of heterotopic ossification (graded using the Brooker classification).

### 2.4. Statistical Analysis

Descriptive statistics were used to summarize demographic data, clinical scores, and radiographic parameters. Continuous variables were expressed as means with standard deviations (SD), while categorical variables were reported as frequencies and percentages. Preoperative and postoperative clinical scores (HHS and WOMAC) were compared using paired *t*-tests, with statistical significance set at *p* < 0.05.

To specifically address the influence of age, the cohort was stratified into two groups according to age at surgery: patients younger than 50 years and those aged 50 years or older. The threshold of 50 years was chosen based on its frequent use in the literature to distinguish younger, more active patients from older individuals undergoing THA. Postoperative HHS and WOMAC scores at the final follow-up were compared between groups using independent-samples *t*-tests. All analyses were performed using SPSS version 25.0 (IBM Corp., Armonk, NY, USA).

## 3. Results

### 3.1. Patients’ Characteristics

A total of 53 patients who underwent Nanos neck-preserving hip arthroplasty between 2008 and 2010 were included in this study. The cohort consisted of 35 men (66%) and 18 women (34%), with a mean age of 50.7 years (range: 35–65 years) at the time of surgery. For subgroup analyses, patients were stratified according to age at the time of surgery: 24 patients were younger than 50 years (mean age 46.5 years), while 29 patients were 50 years or older (mean age 55.5 years).

The predominant indication was primary osteoarthritis (95%), while the remaining cases (5%) included avascular necrosis, developmental dysplasia of the hip (DDH), and post-traumatic sequelae of acetabular fractures (Table 1).

The choice of femoral head size was guided by patient-specific anatomical and functional considerations. The most commonly used femoral head was 36 mm (60% of cases), followed by 32 mm (35%), 28 mm (2%), and 46 mm (3%) (Table 2).

Reporting femoral head size distribution is relevant, as larger heads are associated with increased joint stability, reduced risk of dislocation, and improved range of motion, while also influencing wear rates depending on the bearing couple. In our series, the frequent use of 36 mm heads likely contributed to the very low dislocation rate observed (3.8%).

### 3.2. Clinical Outcomes

**Figure 1:**
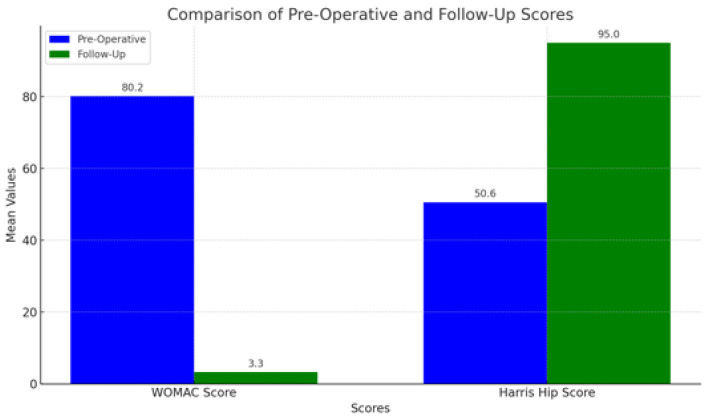
Comparison between preoperative and postoperative clinical scores.

Clinical outcomes were assessed using the Harris Hip Score (HHS) and the Western Ontario and McMaster Universities Arthritis Index (WOMAC). Preoperatively, patients showed marked functional impairment, with a mean Harris Hip Score (HHS) of 50.6 and a mean WOMAC score of 80.2, indicating poor function and substantial pain (Figure 1), (Table 3).

Postoperative scores at 15-year follow-up (Figure 1), (Table 3):

- HHS: Mean score of 95, indicating excellent recovery, significant functional improvement, and minimal pain.
- WOMAC: Mean score of 3.3, suggesting minimal joint pain, stiffness, and functional limitation.

**Figure 2:**
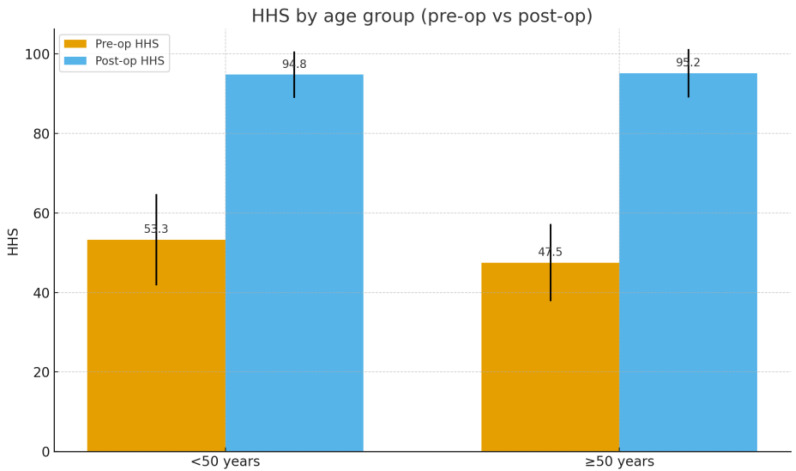
Comparison between preoperative and postoperative HHS by age group.

**Figure 3:**
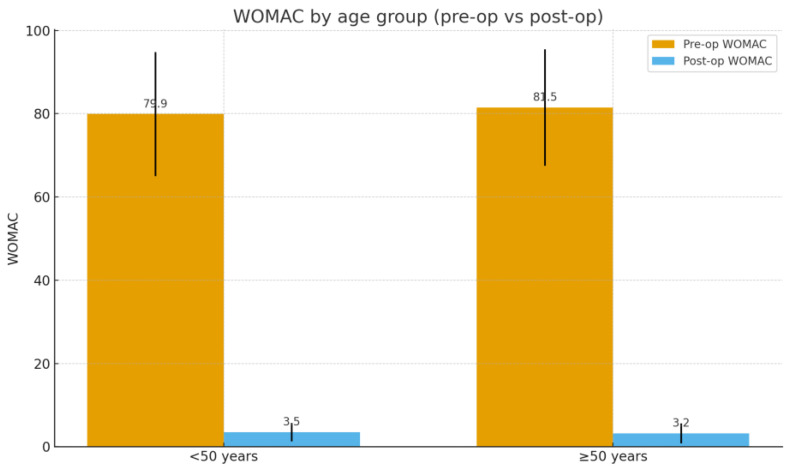
Comparison between preoperative and postoperative WOMAC by age group.

To account for potential age-related differences, the cohort was stratified into two groups: patients younger than 50 years at the time of surgery (*n* = 24, mean age 46.5 years) and those aged 50 years or older (*n* = 29, mean age 55.5 years). Both groups demonstrated comparable preoperative functional impairment (HHS: 53.3 ± 11.5 vs. 47.5 ± 9.7, *p* = 0.056; WOMAC: 79.9 ± 14.9 vs. 81.5 ± 14.0, *p* = 0.705). At final follow-up, outcomes remained excellent in both strata, with mean HHS of 94.8 ± 5.8 in patients <50 years and 95.2 ± 6.1 in those ≥50 years (*p* = 0.72), and mean WOMAC of 3.5 ± 2.2 and 3.2 ± 2.4, respectively (*p* = 0.61) (Figure 2 and Figure 3) (Table 4).

No statistically significant differences were observed between age groups, indicating that long-term functional recovery was consistent regardless of age at surgery.

### 3.3. Radiographic Outcomes

Radiographic analysis focused on implant position, stability, and the presence of osteolysis or heterotopic ossifications.

- Acetabular Cup Position

Acetabular inclination: The mean inclination of the acetabular cup was 44.6°, which is within the optimal range (40–50°) for implant stability and minimal wear risk.

- Implant Stability

Osteolysis: No signs of osteolysis were observed in any of the Gruen zones across the cohort, indicating no bone resorption or implant loosening over 15 years.

Femoral Offset: The mean femoral offset was 46 mm, which reflects a proper restoration of biomechanical parameters. The maintenance of offset is essential for optimal muscle tension and improved joint stability.

**Figure 4:**
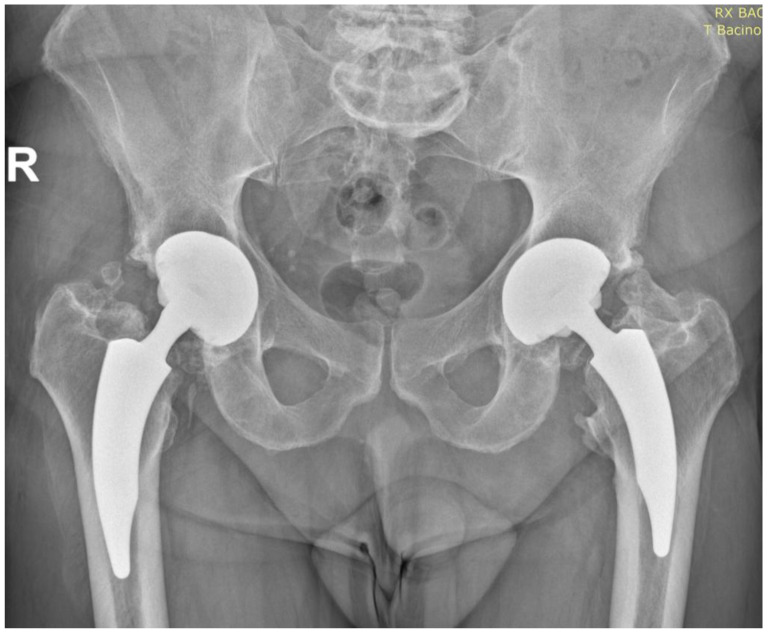
Postoperative AP radiograph at 15 years demonstrating stable fixation of the Nanos^®^ stem and R3™ cup, with heterotopic ossifications.

- Heterotopic Ossifications (Figure 4)

Grade I-II (Brooker classification): Found in 5 patients (9.4%), with no clinical impact or significant functional limitation.

Grade III-IV (Brooker classification): Observed in 2 patients (3.8%), with mild limitation in hip flexion but no pain or other symptoms.

The radiographic outcomes for acetabular inclination, leg-length discrepancy, femoral offset, and heterotopic ossifications are summarized in Table 5.

### 3.4. Postoperative Complications

A total of 3 complications (5.6%) were reported during the 15-year follow-up period. The specific complications are detailed below:

- Dislocation: Two patients experienced postoperative dislocation during bed transfers. Both patients were obese, and the dislocations occurred early in the recovery period. The dislocations were successfully managed with closed reduction, and no further dislocations were reported.
- Cortical perforation: One patient experienced perforation of the lateral femoral cortex during stem implantation. This issue went undetected initially, as the immediate postoperative X-rays were performed only in the AP view, without a lateral projection. The patient, who resided abroad, returned for follow-up after three months. Upon detection of the perforation, the patient underwent a successful revision surgery with the implantation of a Polarstem (Smith & Nephew) primary femoral stem. The postoperative complications and their corresponding details are summarized in Table 6.

**Figure 5:**
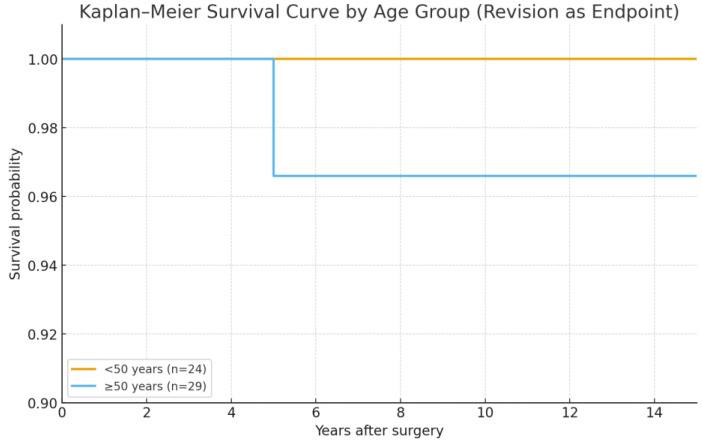
Kaplan–Meier Survival Curve by Age Group (revision and endpoint).

Kaplan–Meier survival analysis, using revision for any reason as the endpoint, demonstrated an overall implant survivorship of 98.1% (95% CI: 94–100%) at 15 years. When stratified by age, patients younger than 50 years (*n* = 24) showed a survival rate of 100%, while those aged 50 years or older (*n* = 29) had a survival of 96.6% (95% CI: 90–100%). No statistically significant differences were observed between groups (log-rank test, *p* > 0.05) (Figure 5).

## 4. Discussion

The NANOS neck-preserving hip prosthesis (Smith & Nephew, Tuttlingen, Germany) demonstrated excellent long-term results in the 53 patients analyzed in this retrospective study. Clinically, patients exhibited significant functional improvement, with an average Harris Hip Score (HHS) of 95 and a low WOMAC score of 3.3 at the 15-year follow-up. These outcomes are consistent with previous reports on short-stem implants, which have shown reliable fixation, preservation of proximal bone stock, and favorable functional results at long-term follow-up [10,11,12,13], without implying superiority over conventional stems.

An advantage of the NANOS prosthesis is its ability to adapt to the patient’s femoral anatomy, allowing for a load distribution that closely resembles natural physiology. Müller et al. highlighted that patients with short-stem prostheses, such as NANOS, exhibit reduced micromovements and improved bone integration. This is particularly advantageous for younger and more active patients who require an implant capable of bearing high loads while preserving as much bone as possible for potential future revisions [5]. Preservation of the femoral neck appears to play a central role in maintaining physiological biomechanics, restoring offset, and facilitating revision surgery when required [14,15,16,17].

An additional relevant finding of our study was the reproducibility of outcomes across different age groups. At final follow-up, patients younger than 50 years improved from a mean HHS of 53.3 to 94.8 and from a WOMAC of 79.9 to 3.5, while patients aged 50 years or older improved from 47.5 to 95.2 (HHS) and from 81.5 to 3.2 (WOMAC). In both strata, within-group paired analyses confirmed highly significant improvements (all *p* < 0.001), indicating that functional recovery and implant survivorship are consistent irrespective of age at the time of surgery. This supports the versatility of the Nanos^®^ stem across a wide patient spectrum.

Although our series did not include an internal control group treated with conventional stems, this limitation was addressed by contextualizing our results within the broader literature. Comparative studies and national registry data consistently report that short stems, including the Nanos design, achieve survivorship and functional outcomes equivalent to conventional stems, while offering the added advantage of bone preservation. By benchmarking our age-stratified outcomes against these external data, the present study confirms that the favorable long-term results of the Nanos prosthesis are not only internally consistent but also aligned with the best available evidence.

A further point of discussion is the comparison between short stems and conventional femoral stems. Although our series lacks an internal control group of conventional THA, several external comparative studies provide valuable context. Steinbrück et al. found no survival difference between short and conventional uncemented stems at mid-term follow-up [41]. Similarly, Díaz-Dilernia et al. reported excellent functional outcomes and comparable implant survival at approximately 10 years in both groups [42]. Large-scale registry data from the Dutch Arthroplasty Register also confirmed no clinically relevant differences in revision risk between uncemented short and standard stems [43]. Taken together, these data reinforce that the favorable long-term outcomes observed with the NANOS stem are in line with broader evidence, indicating that short-stem prostheses perform comparably to conventional implants, while potentially offering the additional advantage of bone preservation.

### 4.1. Advantages of Femoral Neck Preservation

The literature indicates that femoral neck-preserving prostheses are particularly beneficial for younger patients, as preserving bone stock is critical to reducing the risk of revisions, which are more frequent in patients with a longer life expectancy. Preserving the femoral neck maintains bone integrity. It also facilitates easier management of future revisions [19]. Maintaining the native bone structure promotes a more physiological load transfer, thereby reducing the risk of stress shielding and localized bone resorption [3,4]. Bieger et al. demonstrated that short stems significantly minimize stress shielding, improving the longevity of implants and reducing the risk of aseptic loosening [13].

The preservation of trochanteric and femoral neck anatomy also allows for improved rotational stability and biomechanics closer to the native joint, decreasing the likelihood of limb-length discrepancies and malalignment [5,16,18]. These aspects are crucial for younger, more active patients, who often require durable implants capable of sustaining high functional demands. Furthermore, the maintenance of abductor insertions contributes to enhanced joint stability and a lower risk of dislocation [11,18].

Finally, preserving the femoral neck allows for more effective management of revisions in cases of primary implant failure. Morrey et al. highlighted that patients treated with neck-preserving implants experience significantly fewer complications, such as periprosthetic fractures and fixation challenges, compared to traditional implants. This is particularly advantageous for younger patients, who may require easily revisable and durable prostheses. Furthermore, maintaining the femoral neck facilitates the use of traditional revision stems, reducing invasiveness and preserving additional bone for potential future revisions [37].

Other studies, such as that by Mont et al., emphasize that neck-preserving prostheses are ideal for active patients as they allow for optimal functional recovery and superior prosthetic stability over the long term. Preserving the femoral neck also ensures proper muscular alignment, reducing the risk of pathologies associated with the lower limb and improving the overall quality of life for patients [27].

### 4.2. Comparison with Traditional Stems

One of the main advantages of neck-preserving prostheses compared to traditional stems is their ability to conserve a greater amount of native bone, which is critical for long-term implant stability and revision options [7]. Several studies have highlighted the superior bone-preserving characteristics of short stems. For instance, Pongsiri et al. reported long-term survival of short CFP stems over more than 20 years, with significant improvements in HHS [38]. Similarly, Fink et al. observed that short stems reduce the risk of bone resorption and provide favorable outcomes even in younger populations [39].

Another advantage of preserving the femoral neck is the lower incidence of periprosthetic fractures compared to traditional stems. Patel et al. demonstrated that femoral neck preservation helps reduce complications such as stem mobilization and periprosthetic fractures, improving patient comfort and decreasing the need for revisions. Additionally, the reduced invasiveness of the procedure and the preservation of bone stock allow the use of traditional stems without complex bone reconstruction during revisions, an advantageous option for younger patients who may require future revisions [20,35].

Beyond reducing bone-related complications, femoral neck preservation also offers functional benefits. Studies such as that by Santori et al. have shown that maintaining the femoral neck helps preserve the natural biomechanics of the hip, facilitating muscle strength recovery and providing greater joint stability. Preserving the offset and femoral neck length allows for proper alignment of the abductor muscles, reducing the risk of dislocation and improving overall limb function [11].

In our series, patients treated with the NANOS prosthesis reported high satisfaction levels and a low incidence of revisions. The load distribution associated with femoral neck preservation appeared to support stable functional recovery, in line with observations from other short-stem designs, thereby contributing to improved quality of life. This aspect is particularly critical for younger patients, as a more natural force distribution reduces the need for corrective interventions and contributes to greater implant longevity while minimizing the risk of long-term biomechanical complications.

Preserving the femoral neck also allows for maintaining the center of rotation and hip mechanics closer to the native anatomy. As demonstrated by Pipino et al., patients with short stems showed significant improvements in biomechanics. This benefit is essential for reducing limb length discrepancies and muscular dysfunctions that can arise with long stems, improving postural balance and reducing the risk of compensatory pain in the long term. Additionally, femoral neck preservation contributes to improved implant stability, as highlighted by Nawabi et al., who reported a lower incidence of aseptic loosening and longer prosthetic longevity in patients treated with short stems [9,11].

Finally, femoral neck preservation offers flexibility in revision management, allowing the use of traditional or short stems without requiring additional femoral bone removal. Grappiolo et al. reported that revisions in patients with short stems are generally less complex than those with traditional stems, thanks to the lower invasiveness of the initial implant and the preservation of the trochanter and femoral neck. This advantage is particularly significant for younger patients, who may benefit from less invasive implants and simpler revisions in cases of primary implant failure [17].

In addition to these results, external comparative studies directly contrasting short and conventional stems provide valuable context. Kim et al. [44] compared ultrashort and conventional anatomic cementless femoral stems in patients younger than 55 years, reporting excellent survivorship and comparable functional outcomes at mid- to long-term follow-up. Steinbrück et al. found no survival differences between short and conventional uncemented stems at mid-term follow-up [41]. Díaz-Dilernia et al. confirmed comparable outcomes at 10 years [42], while van Veghel et al., using data from the Dutch Arthroplasty Register, demonstrated equivalent revision risks between the two designs [43].

**Figure 6:**
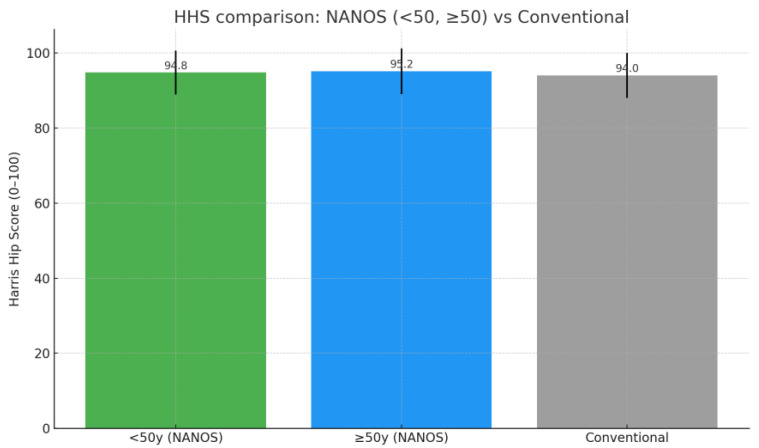
WOMAC Comparison: NANOS vs. traditional stem [44].

**Figure 7:**
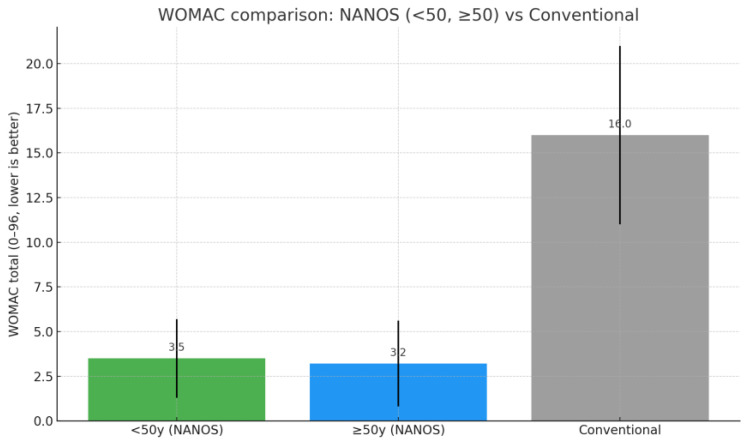
HHS Comparison: NANOS vs. traditional stem [44].

To further strengthen this comparison, we analyzed our results stratified by age groups (<50 years and ≥50 years) against published benchmarks for conventional stems. As illustrated in Table 7 and Figure 6 and Figure 7, postoperative HHS was excellent in both subgroups (94.8 ± 5.8 in patients <50 years; 95.2 ± 6.1 in those ≥50 years) and comparable to values reported for conventional cementless stems in young patients from the study by Kim et al. [44]. WOMAC scores at final follow-up were similarly favorable in both NANOS subgroups (3.5 ± 2.2 vs. 3.2 ± 2.4), and distinctly lower than those typically reported for the conventional stem group (16.0 ± 5.0) [44]. These findings confirm that short stems, including the NANOS design, achieve outcomes at least equivalent to conventional stems, while offering the additional advantage of bone preservation and simpler revision options.

### 4.3. Application in Younger Patients

The increasing demand for THA among younger and more active patients has raised the importance of bone-preserving solutions. Short-stem prostheses such as the NANOS allow substantial preservation of femoral bone, facilitating less invasive revisions if needed in the future [19,32]. This aspect is crucial in patients expected to undergo multiple surgeries during their lifetime. Previous studies have confirmed that short stems maintain stability and clinical function in younger cohorts, with survival rates above 90% even after more than a decade [5,31,32].

By preserving the femoral neck and optimizing load transfer, these implants ensure rapid recovery, reduced thigh pain, and lower incidence of biomechanical complications such as limb-length discrepancy or reduced offset [7,9,11]. These advantages are particularly relevant for young, active patients, who typically place higher functional demands on their implants [40].

### 4.4. Surgical Technique Precision

While the NANOS hip prosthesis offers several advantages, its surgical technique requires substantial expertise. Precise preparation of the femoral canal and accurate stem positioning are crucial to avoid complications such as malalignment, cortical perforation, or periprosthetic fractures [8,17,45]. Once the learning curve has been overcome, however, outcomes are excellent, with high reproducibility and low complication rates [8,46].

Technological advances, such as navigation systems and 3D imaging, further improve the accuracy of stem positioning and reduce technical errors [24,47]. Preservation of native femoral anatomy not only enhances biomechanical stability but also contributes to faster functional recovery and reduced rehabilitation times [12].

Nevertheless, several limitations of the Nanos design should be acknowledged. Neck-preserving stems demand higher surgical precision compared to traditional designs, particularly during the learning curve, which increases the risk of cortical perforation or leg-length discrepancies, as also reflected by the complication observed in our series [8,17]. Moreover, the applicability of the Nanos stem is restricted in patients with severe metaphyseal deformities, marked osteoporosis, or extended bone loss, where conventional or longer stems remain more reliable options [19,20]. Lucero et al. [48] specifically identify severe deformity, osteoporosis, and mismatches between femoral neck and metaphyseal geometry as contraindications for short-stem implantation.

Regarding long-term revision strategies, the theoretical advantage of easier revision due to bone preservation remains to be confirmed by comparative data. Mauch et al. [49] reported that short stems can be used in revision THA in patients with good bone quality, but complication risks, such as intraoperative fractures, remain higher compared to standard stems.

Evidence from long-term series of other neck-preserving stems provides useful benchmarks. Piakong et al. [32] reported Kaplan–Meier survivorship of the CFP stem of 93.2% at 5 and 10 years and 83.0% at 20 years. Similarly, Stauss et al. [50] demonstrated 95.5% survivorship at 15 years with the Metha^®^ stem in a large cohort of over 1300 patients. These results confirm that short stems can achieve outcomes comparable to conventional stems, though superiority cannot be claimed without direct head-to-head trials. In our cohort, Kaplan–Meier analysis demonstrated an overall 15-year survivorship of 98.1%. When stratified by age, survival was 100% in patients younger than 50 years and 96.6% in those aged 50 years or older, with no statistically significant difference between groups. These findings reinforce the long-term safety and durability of the Nanos stem, while remaining consistent with outcomes reported for other short-stem designs.

Finally, in specific scenarios such as young osteoporotic patients with Dorr type C femora, short-stem implantation has been reported as feasible, though with strict selection criteria and limited evidence. Zhen et al. [51] demonstrated encouraging mid-term outcomes in this challenging subgroup, but emphasized the need for careful patient selection.

Taken together, these considerations underline that while the Nanos prosthesis can provide durable and reproducible outcomes, its indications must be carefully individualized, and longer-term comparative studies are required to validate the hypothesized revision advantages and biomechanical benefits [19,32,37].

### 4.5. Limitations and Bias

This study presents some limitations inherent to its retrospective design, including potential selection and reporting biases. The relatively small sample size reduces statistical power and, together with the lack of a control group, limits the generalizability of the findings. All procedures were performed by a single experienced surgeon, which ensured technical consistency but may not reflect outcomes in less specialized settings. Moreover, the absence of a standardized radiographic protocol across all follow-ups may have introduced variability in imaging interpretation. The lack of an internal control group of conventional stems prevents direct comparisons, although this limitation was mitigated by benchmarking against published literature and registry data. In addition, the surgical technique required for Nanos stem implantation is technically demanding, particularly during the learning curve, and may not be easily reproducible in all settings. Despite these limitations, the study provides valuable long-term data on the performance of the Nanos neck-preserving stem in a well-defined patient cohort.

## 5. Conclusions

This study demonstrated excellent long-term clinical and radiographic outcomes with the Nanos^®^ hip prosthesis at 15 years, with high survivorship and patient satisfaction. Despite the limitations of the retrospective design and small sample size, our results confirm that the Nanos stem is a reliable bone-preserving option for younger and active patients. It should be regarded as one of several neck-preserving short stems currently available, all sharing the common goal of conserving bone stock and facilitating future revision surgery. Data from multicenter studies and national registries further support the role of short-stem designs as valid alternatives to conventional implants in contemporary total hip arthroplasty.

## Figures and Tables

**Table 1:** Etiology of pathology of patients treated in the study.

Etiology	
Primary osteoarthritis	95%
Avascular necrosis	5%
Development dysplasia	Included in 5%
Acetabular fracture sequelae	Included in 5%

**Table 2:** Femoral head side of treated patients included in the study.

Femoral Head Size	
28 mm	2%
32 mm	35%
36 mm	60%
46 mm	3%

**Table 3:** Clinical outcomes (preoperative vs. follow-up).

Clinical Parameters	Preoperative (Mean)	Postoperative (Mean)
HHS	50.6	95
WOMAC Score	80.2	3.3
Leg Length Discrepancy	-	2.7 mm
ROM	Limited	Full
Squeaking	Not assessed	3 patients (5.6%)

**Table 4:** Clinical outcomes stratified by age group (preoperative vs. follow-up).

Age Group	*n*	Preoperative HHS (Mean ± SD)	Postoperative HHS (Mean ± SD)	Preoperative WOMAC (Mean ± SD)	Postperative WOMAC (Mean ± SD)
<50 yrs	24	53.3 ± 11.5	94.8 ± 5.8	79.9 ± 14.9	3.5 ± 2.2
>50 yrs	29	47.5 ± 9.7	95.2 ± 6.1	81.5 ± 14.0	3.2 ± 2.4

**Table 5:** Radiographic Outcomes.

Radiographic Parameters	Value
Acetabular inclination	44.6°
Leg Length Discrepancy	2.7 mm
Femoral offset	46 mm
Osteolysis (Gruen zones).	None
Heterotopic Ossification (Grade I–II)	5 patients (9.4%)
Heterotopic Ossification (Grade III–IV)	2 patients (3.8%)

**Table 6:** Postoperative complications.

Type of Complication	Cases	Details
Dislocation.	2	Occurred during transfers in obese patients; managed with closed reduction
Cortical perforation.	1	Lateral femoral cortex perforation; patient revised with Polarstem Smith&Nephew

**Table 7:** Groups comparison: NANOS <50, NANOS >50 vs. traditional stem [44].

Group	HHS (mean ± SD)	WOMAC (mean ± SD)
<50 years	94.8 ± 5.8	3.5 ± 2.2
>50 years	95.2 ± 6.1	3.2 ± 2.4
Conventional [44]	94.0 ± 6.0	16.0 ± 5.0

## Data Availability

The datasets used and/or analyzed during the current study are available from the corresponding author on reasonable request.

## Abbreviations

The following abbreviations are used in this manuscript:

THA	Total Hip Arthroplasty
HHS	Harris Hip Score
WOMAC	Western Ontario and McMaster Universities Arthritis Index
ROM	Range of Motion
LLD	Leg Length Discrepancy
DDH	Developmental Dysplasia of the Hip
IRB	Institutional Review Board
RCT	Randomized Controlled Trial
SD	Standard Deviation
CI	Confidence Interval
